# Chronologically matched toenail-Hg to hair-Hg ratio: temporal analysis within the Japanese community (U.S.)

**DOI:** 10.1186/1476-069X-11-81

**Published:** 2012-10-31

**Authors:** Thomas Hinners, Ami Tsuchiya, Alan H Stern, Thomas M Burbacher, Elaine M Faustman, Koenraad Mariën

**Affiliations:** 1 National Exposure Research Laboratory, U.S. Environmental Protection Agency, Las Vegas, NV, USA; 2 Department of Environmental and Occupational Health Services, University of Washington, Seattle, WA, USA; 3 Department of Environmental Protection, Trenton, NJ, USA; 4 Institute for Risk Analysis and Risk Communication, University of Washington, Seattle, WA, USA; 5 Department of Health, Olympia, WA, USA

**Keywords:** Mercury, Methylmercury, Cardiovascular, Hair, Toenail, Chronological, Biomarker

## Abstract

**Background:**

Toenail-Hg levels are being used as a marker of methylmercury (MeHg) exposure in efforts to associate exposure with effects such as cardiovascular disease. There is a need to correlate this marker with more established biomarkers that presently underlie existing dose–response relationships in order to compare these relationships across studies.

**Methods:**

As part of the Arsenic Mercury Intake Biometric Study, toenail clippings were collected at three time points over a period of one year amongst females from within the population of Japanese living near Puget Sound in Washington State (US). Variability in temporal intra-individual toenail-Hg levels was examined and chronologically matched hair and toenail samples were compared to more accurately define the toxicokinetic variability of Hg levels observed between the two compartments.

**Results:**

Mean toenail-Hg values (n=43) for the 1^st^, 2^nd^ and 3^rd^ visits were 0.60, 0.60 and 0.56 ng/mg. Correlations were as follows: r=0.92 between 1^st^ and 2^nd^ clinic visits, r=0.75 between 1^st^ and 3^rd^ visits and r=0.87 between 2^nd^ and 3^rd^ visits. With few exceptions, toenail-Hg values from any visit were within 50-150% of the individual’s mean toenail-Hg level. Nearly all participants had less than a two-fold change in toenail-Hg levels across the study period. A regression model of the relationship between toenail-Hg and hair-Hg (n = 41) levels representing the same time period of exposure, gave a slope (Hg ng/mg) of 2.79 for hair relative to toenail (r=0.954).

**Conclusions:**

A chronologically matched hair-Hg to toenail-Hg ratio has been identified within a population that consumes fish regularly and in quantity. Intra-individual variation in toenail-Hg levels was less than two-fold and may represent dietary-based fluctuations in body burden for individuals consuming various fish species with different contaminant levels. The chronologically matched ratio will be useful for relating MeHg exposure and dose–response derived from toenail-Hg measurements to those derived from hair-Hg measurements in other studies, and may be useful in future investigations as an indicator of stable MeHg body burden within a population.

## Background

As most human exposures to methylmercury (MeHg) result from fish consumption, federal and state consumption advisories have been released as a public health protective measure [1,2]. Most state and federal advice to fish consumers reflects consideration of the US EPA reference dose (RfD) for MeHg [3]. The RfD, as well as the general recognition of the hazardous nature of MeHg exposure is in turn based upon extensive neurological research that has been repeatedly summarized e.g., [4-6]. In addition to concerns over neurophysiological deficits that can occur in children exposed *in utero* to MeHg, concern has been raised about the possible role of MeHg in cardiovascular disease in adults. There have been several major epidemiological studies conducted that investigated the relationship between MeHg exposure and the incidence of myocardial infarction [7-11]. The findings from four of these five studies along with additional data relating MeHg exposure with, for example, heart rate variability and oxidative stress were critical to a panel assembled under contract to the US EPA that recommended the development of a dose–response function for myocardial infarction and MeHg exposure in a regulatory benefits analyses addressing Hg emissions [12]. Roman et al. [12] did not address findings from the subsequently published fifth study which did not observe an association between MeHg exposure and coronary heart disease or stroke [11].

Hair-Hg, blood-Hg and toenail-Hg levels have been used as biomarkers of exposure for MeHg with each biomarker having been significantly correlated with fish consumption which is the primary source of MeHg exposure [4,6,13-15]. Analyses of hair-Hg and blood-Hg levels in fish consumers and non-fish consumers have been conducted allowing for these compartments to be used to characterize exposure to MeHg within a population [4]. Hair- and blood- Hg concentration have been the biomarkers of MeHg exposure used most frequently in studies investigating the relationship between MeHg exposure and neurodevelopmental outcomes as well as in several of the major studies investigating cardiovascular effects [7,10,16-18]. However, several important epidemiological studies investigating the relationship between exposure and cardiovascular outcomes have relied on toenail-Hg levels as the biomarker for MeHg exposure [8,9,11]. Data on the use of toenail-Hg levels as a biomarker of exposure is less robust compared to data from hair and blood compartments. The foundation of the toenail compartment as a biomarker is primarily based on observed correlations with hair-Hg levels and fish consumption with several studies having assessed MeHg exposures using toenails and a few having compared measurements across multiple markers [13-15,19-26]. Several studies within this group have provided for data allowing hair-Hg to toenail-Hg ratios to be established based on mean values and have developed regression models examining the relationship between hair-Hg and toenail-Hg levels [13,15,21,22,26]. However, despite these available data comparing Hg concentrations between two compartments, data are not available comparing hair and toenail biomarker results from exposure that occurred over the same time period.

As part of the Arsenic Mercury Intake Biometric Study (AMIBS), we compared Hg levels from hair segments and toenail clippings reflecting the same time period of exposure and examined longitudinal data for toenail-Hg levels obtained at three time points within the population of Japanese women living in the Puget Sound area of Washington State (US). Previous work has examined fish intake and MeHg body burden in this population and results indicated that this Japanese population consumes substantially more fish than the national average and that MeHg exposure within this group is among the highest in the US [27,28]. The goals of this work were two-fold: to more accurately define the toxicokinetic variability of Hg levels observed between the hair and toenail compartment, and to examine variability in temporal toenail-Hg levels that were obtained over a period of approximately one year. This information will permit an improved estimation of MeHg exposure based on the toenail compartment which is used as an exposure metric in studies examining the relationship between cardiovascular effects and MeHg exposure and may be useful in future studies examining the relationship between neurological effects and MeHg exposure. It will also facilitate the comparison between the results of studies employing toenail- and hair-Hg levels as biomarkers of exposure.

## Methods

### AMIBS

As a continuation of AMIBS, hair and toenail data were collected from a subset of the 106 Japanese women participants. The participants were sampled three times across 14 months and this manuscript describes the results from 43 women who provided toenail samples during all three clinic visits and 41 women who provided a toenail and hair sample whose length allowed chronological matching with a toenail sample. Chronologically matching biomarker samples allowed for the same exposure period to be compared. AMIBS has been previously described in detail [27,28]. Briefly, the study consisted of women of childbearing age (18 – 45 years of age), who identified themselves as Korean, Japanese or, of Japanese or Korean descent, and who had lived in the Puget Sound area of Washington State, US, for at least 6 months. The AMIBS group consisted of 214 women (108 Korean and 106 Japanese participants). Participation in the study required that the women provide a hair sample from the nape of the neck (0.5 cm in diameter) and complete a fish consumption survey. Participants also completed a self-administered food frequency questionnaire and could provide additional biological samples (urine, blood and/or toenails) at their discretion.

Informed consent was obtained from all study participants, and the study design and materials were approved by the State of Washington Department of Social and Health Services Human Research Review Board.

### Hair and toenail sampling

To assess growth rates for hair and toenail, the capillary-tube method and the etching method were applied, respectively [29,30]. Hair growth rate determinations using the method described by Saito et al. [29] are provided with some frequency in the literature, yet the method described by Dawber [30] of etching the nail for determining toenail growth rates is less often provided. Toenail growth rate was determined by mechanically etching a groove in the nail plate at the highest point of the lunula perpendicular to the direction of nail plate growth during the participant’s first clinic visit. After etching the nail plate, the distance from the top of the lunula to the eponychium was measured. Upon return visit, the distance between the marking and the top of the lunula as well as between the marking to the eponychium were measured. As both the size and shape of lunula and the eponychium can change slightly over time, the average of both measurements was used along with the time span between visits to determine growth rates.

Toenail growth rates for 35 of the 43 women participants and hair growth rates for 29 of the 43 women participants were obtained during the study period. Individual growth rates were used to determine the date at which the nail emerged from beneath the eponychium as well as when the hair initially protruded from the scalp. For example, by dividing the length of the nail from the eponychium to the distal end of the nail by nail growth rate, the number of days since the nail protruded from beneath the eponychium was obtained.

The time point of exposure identified by the distal end of the toenail clipping, which represents the oldest portion of the nail, was used to determine which hair segment (from hair strands obtained at an earlier clinic visit) was required for analysis. The hair segment was identified based on the date the hair strands were obtained (cut from the nape of the neck) and the individual’s hair growth rate value. With the same time point of exposure identified on the nail and hair segment, chronologically matching samples were acquired for analyses. For those 8 and 14 women, respectively, for whom toenail and hair growth rates were not obtained, the average growth rates from those individuals for whom data were available, were used in calculations to obtain chronologically equivalent samples.

In general, the axial lengths of toenail clippings were between approximately 1mm and 3mm. These toenail clippings reflect not just the point in time defined by the distal end of the clippings, but represent a few weeks of growth. Accordingly, the hair sample segment was specifically identified to encompass the exposure period of a toenail clipping as defined from the distal to the proximal end of the clipping (the axial length). The length of the hair segment sample analyzed represented the window of exposure on the toenail identified as being between:

1) The distal end of the toenail clipping to a time point 60 days prior (which on the toenail is equivalent to 60 days of growth in the direction of proximal end of clipping and exceeds the time period identified by the axial lengths of toenail samples), and

2) The distal end of the toenail clipping to a time point 30 days beyond the exposure time point identified by the distal end of the toenail clipping.

Accordingly, from the location on the hair strand considered to be chronologically equivalent to the time point identified by the distal end of the toenail sample, a hair segment was analyzed that included 30 days growth in the direction of the distal end of the hair segment as well as a period of growth representing 60 days towards the proximal end of the hair segment. In total, a period of approximately 90 days exposure was reflected by the hair segment so as to insure that the time period of exposure represented by the entire toenail clipping was included within the hair segment length analyzed.

The hair segment representing an exposure window of 90 days was chosen so as to encompass the period of exposure identified by the axial length of the toenail sample as well as to address areas of uncertainty associated with uptake of Hg into hair and toenail. Although we assume that the incorporation of Hg into the nail matrix and hair follicle is directly proportional to the level of Hg in blood, there were several factors identified that resulted in a hair segment encompassing an exposure window exceeding that produced by the toenail sample. First, there are no available data regarding the time required between MeHg intake and uptake into the nail root and nail matrix so as to become visible at the eponychium. Second, the time delay between uptake of Hg from blood and into the hair shaft that protrudes above the scalp has been estimated to be between 20 and 60 days [23,31,32]. Third, the axial thicknesses of the toenail samples varied between individuals and it varied within each individual’s sample (i.e., due to tapering of the toenail clippings at each end, the axial thickness of each clipping was not uniform). This lack of precision in toenail clipping samples as well as uncertainty in Hg uptake into the biological markers, did not make it feasible to determine the exact exposure period encompassed between the distal and proximal end of the toenail clipping nor did it allow for determination of the precise position along the hair strand reflecting the time point associated with the distal end of the toenail clipping. Accordingly, a segment of hair growth was analyzed considered to encompass the time period of exposure represented by the toenail clipping.

### Hair- and toenail-Hg analyses

Specimens of hair (0.5-cm diameter bundles from the nape of the neck) and of protruding nail (from both big toes) were collected at three visits. Each chronological hair segment matching the toenail sample was obtained from strands individually cut after being suspended between two self-closing bent forceps and viewed under 1.75x magnification. Accuracy of hair-strand cutting was ensured with the use of suture (stitch) scissors (where the wider notched blade, designed to lift the suture, allowed close positioning to the hair strand without premature cutting) in combination with resting the notched scissor blade in the desired-length groove of a stainless-steel ruler, which permitted the magnified viewing to be unceasingly focused on aligning the free-end of the fiber until the scissor blades were closed. Segments not cut within 0.02 cm of required length (as viewed under 4.25x magnification) were rejected to minimize segmenting as a variable. For each hair sample, chronological hair segments from several fibers were combined to provide at least 1.00 mg (see Additional file 1 for a discussion of hair-Hg representativeness). Chronological hair segments for nearly all the participants were obtained within the first 8 cm from the proximal end of the hair strand.

Toenail clippings were obtained from the big toes using stainless steel scissors. The big toe was selected because the nail beds differ in length among the toes; this results in the available nail segments on each toe having been formed at different times. Clippings were stored in labeled micro-centrifuge tubes, and transported in plastic bags. The toenail clippings were wiped with an alcohol pad (individually packaged) to remove any adhering dermal tissue and dried to constant weight at ambient conditions. Hg analyses for hair and toenails were conducted as described previously using a direct analyzer (Milestone, Inc., Shelton, CT) following EPA Method 7473 [28,33]. The instrument detection limit was near 0.01 ng (corresponding to 0.001 ng/mg for a 10 mg hair or toenail sample). Quality assurance included the measurement of reference materials before and after sample analyses and, participation in Health Canada Mercury-in-Hair Interlaboratory Comparison Program [34]. Because our QC plan specified that Hg results for reference materials that exceeded ±10% of the nominal Hg mean concentration (adjusted for the moisture content) would be used to normalize the data from samples obtained during the same measurement session, raw data for the reference materials (primarily Albacore Tuna, NBS RM 50) do not reflect the accuracy for the samples. Using this calibration-normalization procedure, our blind results for 21 specimens from the Health Canada Mercury-in-Hair Interlaboratory Comparison Program were 100.4 ± 2.9% (mean ± SD) of the consensus Hg means (range 0.92 to 10.05 μg/g, median 4.66 μg/g) from 19 to 25 laboratories. Our measurement precision was sufficient to distinguish (p <0.001) between two Health Canada specimens that differed in Hg by only 3% (2.35 vs 2.28 μg/g). Individual toenail-Hg concentrations from left and right foot were averaged for this study as the relative percent difference (RPD) between individual left and right toenail mercury levels was small (RPD = 6.5%, range 0.1 to 16%).

### Statistical analyses

A repeated-measures one-way ANOVA was used to compare toenail-Hg levels among sample intervals. Student’s *t*-test was applied to compare means of toenail-Hg levels as well as hair-Hg levels based on pregnancy status. Pearson product–moment correlation coefficients were determined to compare toenail-Hg levels with hair-Hg values. Statistical analyses were performed using a significance level of 5% (p < 0.05) using Stata (Stata Corporation, College Station, Texas), Excel (Microsoft Corporation, Redmond, Washington), VassarStats (http://faculty.vassar.edu/lowry/VassarStats.html) and SPSS (SPSS Inc., Chicago, Illinois).

## Results

**Figure 1:**
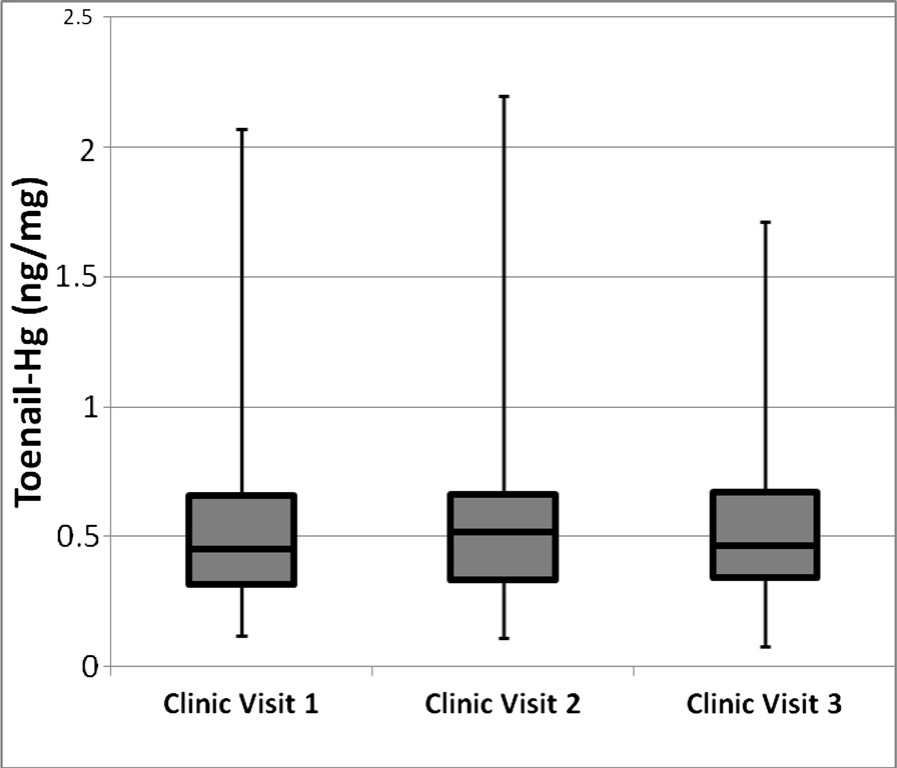
**Toenail-Hg levels for each visit (n = 43).** Medians are middle lines within box. Top and bottom of box represents upper and lower quartile values, respectively. Upper and lower whiskers represent sample maximum and minimum values, respectively. Distribution mean values do not differ significantly (p <0.05).

Toenail and hair samples were collected from 43 Japanese participants across three clinic visits. Mean toenail growth rate was 0.048 ±0.012 mm/day (22.4 ±6.5 days/mm) and mean hair growth rate was 0.42 ±0.07 mm/day (24.5 ±5.3 days/cm). No significant difference in toenail-Hg or in hair-Hg levels was observed between pregnant (n = 11) and non-pregnant participants (n = 32) (p > 0.05). The average number of days between visits was 135 (± 18) days with the shortest interval being 100 days and the longest, 189 days. The interquartile range was 126 to 141 days. The mean toenail growth length across the average number of days between visits was 6.48 mm (135 days x 0.048 mm/day). The mean time period spanned by the three visits was 270 days (range, 226 to 350). Interquartile Hg-concentration ranges for the three visits were 0.33-0.67, 0.34-0.66 and 0.34-0.67 ng/mg, respectively. Respective 95^th^ percentile confidence intervals were ± 0.12, 0.13 and 0.10. Arithmetic mean toenail-Hg values with standard deviations and coefficients of variation in parentheses for the 1^st^, 2^nd^ and 3^rd^ visits were 0.60 ±0.42 (0.70), 0.60 ±0.43 (0.72) and 0.56 ±0.35 (0.63) ng/mg with box plots depicting visit distributional data provided in Figure 1. No significant difference in toenail-Hg levels was observed among the three sampling visits, but the relatively small number of samples limited the power to detect a small difference. Pearson’s product–moment correlation coefficients of Hg concentration between sampling intervals were as follows: r = 0.92 between 1^st^ and 2^nd^ clinic visits, r = 0.87 between 2^nd^ and 3^rd^ visits and r = 0.75 between 1^st^ and 3^rd^ visits.

**Figure 2:**
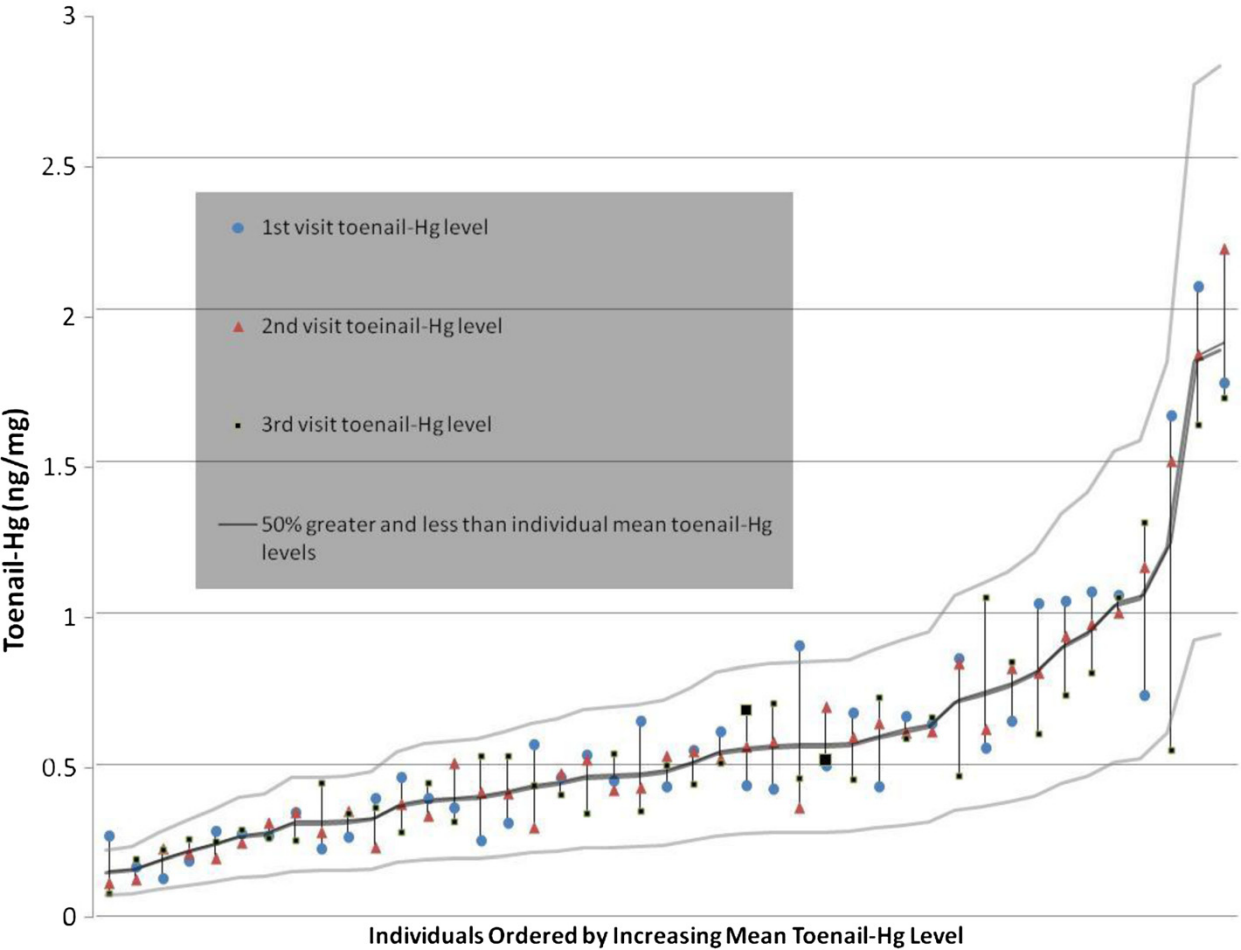
Intra-individual toenail-Hg variability across three successive clinic visits.

**Figure 3:**
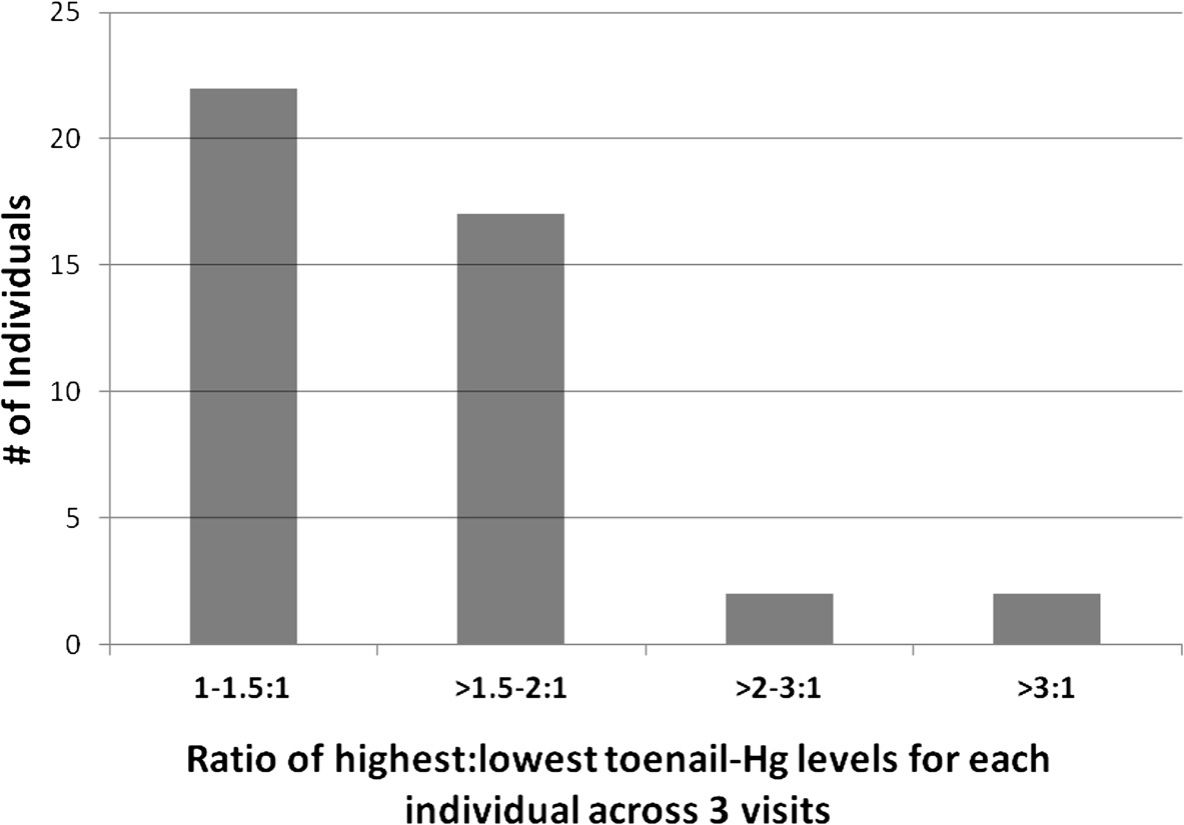
Ratio of intra-individual toenail-Hg levels (n = 43).

The absolute change in toenail-Hg levels on an individual basis between successive samples across the three visits is depicted in Figure 2. With the exception of three toenail-Hg levels (out of 129), all values for separate clinic visits were within 50% to 150% of the individual’s mean toenail-Hg level. The extent of intra-individual change among participants depicted as the ratio of highest to lowest toenail-Hg levels from the visits for each individual is provided as a histogram in Figure 3. Nearly all participants (39 of 43) had less than a two-fold change in toenail-Hg levels across the one-year time period encompassed by the three samples with half (22 of 43) not exceeding a 1.5-fold change. Range of change was 1.08 - 3.44.

**Figure 4:**
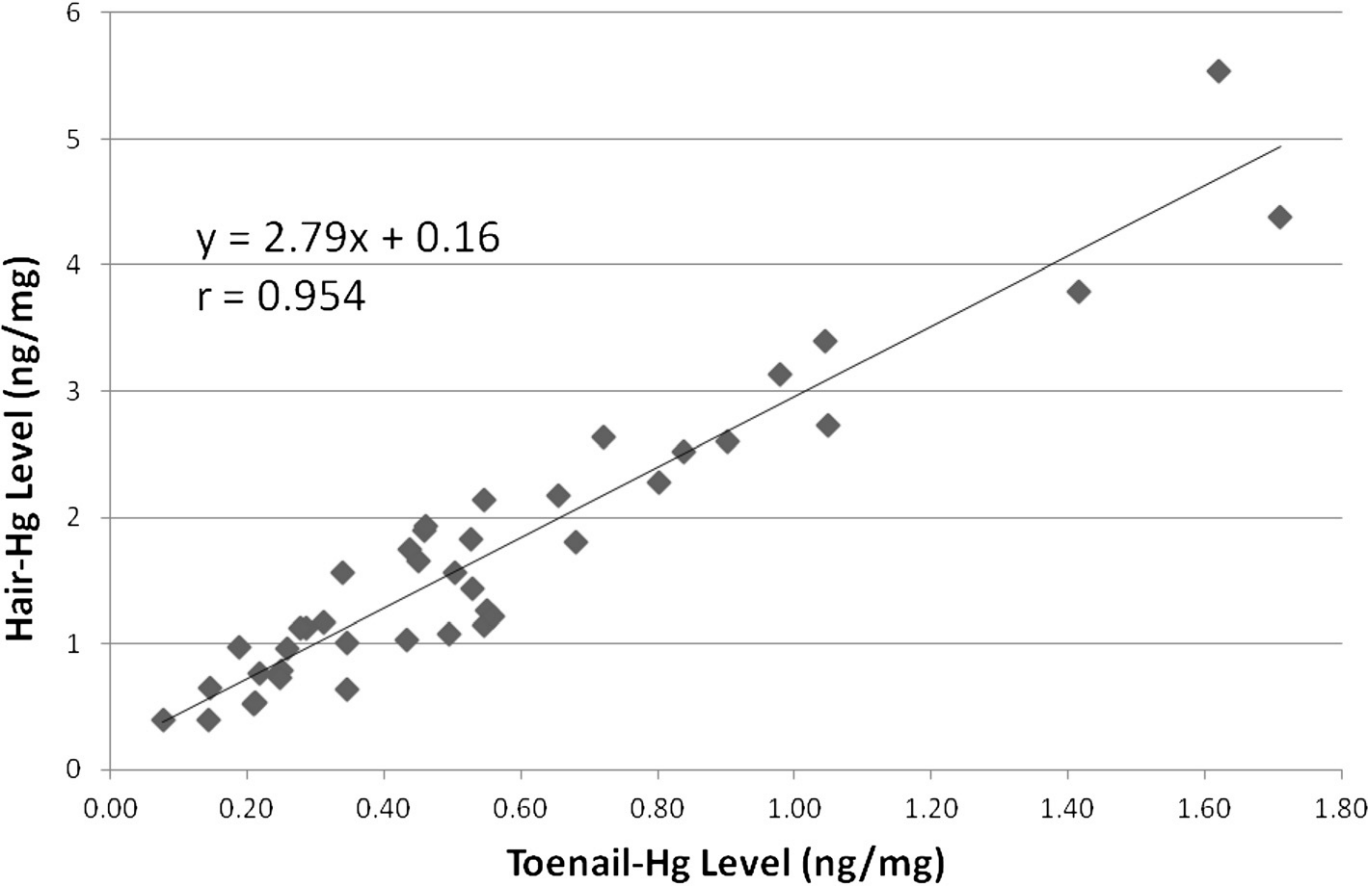
Relationship between toenail- and hair- Hg levels (ng/mg) among participants (n= 41).

Chronologically matched sample evaluation was conducted by comparing toenail-Hg levels with hair-Hg values representing the same time period of exposure (n = 41). Chronological matching of samples was not possible for all individuals due to factors such as timing of sample collections and length of hair samples. A regression model of the relationship between chronologically matched toenail-Hg and hair-Hg samples reflecting the same period of exposure provided for a slope (Hg ng/mg) of 2.79 for hair relative to tonail (r=0.954) (Figure 4). For purposes of comparison with previously conducted studies, the regression line of hair-Hg on toenail-Hg using results from samples obtained at the same point in time (i.e. during a clinic visit) and thus not reflecting the same window of exposure (chronologically unmatched) was determined and yielded a slope of 2.39 (r = 0.871)(data not shown). A comparison of results on group mean biomarker-Hg levels identified from the literature are provided in Table 1. The table gives ratios of hair-Hg to toenail-Hg based on study mean values with the addition of slope values from regression models of hair-Hg on toenail-Hg provided from three of the studies [13,15, this study]. The ratio of hair- to toenail-Hg based on arithmetic mean values for this study was 3.08 and 2.77 based on chronologically matched and unmatched samples respectively. The unmatched sample ratio of 2.77 was similar to the ratio (2.56) obtained by Ohno and co-workers [15] within a similar population (non-occupationally exposed group of Japanese women) and also similar to ratios obtained from a control group (2.4) in a cohort study of dentists as well as from one of two cohorts (2.5) studied in Finland [13,26]. The slope from the regression model using chronologically unmatched sample results (2.39) was similar to the slope (2.44) obtained by Ohno and co-workers [15] and by Alfthan [13] in one of two cohorts (2.24). For comparative purposes, a ratio of 3.16 was derived and is presented in Table 1 using geometric mean values for hair-Hg and toenail-Hg from matched samples; 1.41 ng/mg and 0.45 ng/mg, respectively.

**Table 1:** Hair-Hg to toenail-Hg ratios determined from regression models and mean values obtained from literature

**Hair-Hg: Toenail-Hg**			**n**	**Population**	**Citation**
Based on arithmetic means	Based on regression model	Based on geometric means			
4.5:1^a^	4.2:1^a^		30 (hair), 29 (toenail)	Non-occupationally exposed Norwegians^b^	[21]
		3.58:1	42	Faroese whaling men	[22]
2.56:1	2.44:1^c^		59	Non-occupationally exposed Japanese women	[15]
2.8:1	2.24:1^c^	2.5:1	72	Non-occupationally exposed Finns	[13]
4.3:1	4.21:1^c^	3.5:1	39		
1.4:1			161 (hair), 163 (toenail)	Dentists	[26]
2.4:1			161 (hair), 155 (toenail)	Controls	
3.08:1	2.79:1^c^	3.16:1^d^	41 (chronologically matched samples)	Non-occupationally exposed Japanese women (U.S.)	this study
2.77:1	2.39:1^c^		41 (unmatched samples)		

a. Study data provided for blood-Hg and toenail-Hg values along with regression model. Hair-Hg to blood-Hg ratio of 250:1 was used to obtain hair-Hg: toenail-Hg and slope of regression model of blood-Hg on toenail-Hg [4].

b. One individual had previous occupational exposure as a dental nurse.

c. Ohno and co-workers [15] and Alfthan [13] provided regression models of hair-Hg on toenail-Hg with resulting slope factors. Slope in this study from linear regression model based on chronologically matched and unmatched samples are provided.

d. For this study, geometric mean values for hair-Hg and toenail-Hg from matched samples were 1.410 ng/mg and 0.446 ng/mg, respectively.

## Discussion

Toenail clippings from females were examined over a period of approximately one year to investigate variability in temporal intra-individual toenail-Hg levels, and chronologically matched hair and toenail samples were compared to more accurately define the toxicokinetic variability of MeHg levels observed between the two compartments.

Hair-Hg levels from this population of females have previously been investigated with results showing no significant change in MeHg body burden levels across the study period suggesting that if MeHg population-based body burden levels were changing they were doing so slowly and across a time period larger than the one investigated [35]. This study corroborates that conclusion and further, indicates that while there was temporal stability of the three toenail-Hg levels for the group as a whole, individuals had variable MeHg body-burden levels but the levels did not fluctuate by more than two-fold across the one-year period investigated. The fluctuation in individual body burden levels is likely due to changes in MeHg intake across sample intervals while intra-individual toxicokinetic variability may also be a contributing factor. The intra-individual change in Hg levels observed across the study period which did not exceed 1.5-fold for half of the participants and two-fold for nearly the entire study group can be considered normal behavioral variability that is non-directional fluctuation within longer term temporal stability. For individuals with stable body burden levels, a single data point value, such as toenail-Hg levels obtained at a given time, could be used to reflect long term MeHg body burden; with the individual’s mean value being within the interval defined by one-third to twice the obtained toenail-Hg level. It must be noted however, that for identifying body burden levels across a short time period, even within those having temporal stability, biomarker based measurements from a single time point used to estimate exposure can miss short term elevated MeHg exposure periods consisting of single exposures to those lasting weeks [36-38].

For toenails to be used as a biomarker for long term exposures such as years, there is a need to either verify temporal stability within the study population by comparing biomarkers reflecting body-burden levels against an empirically derived ratio as defined herein, or repeated biomarker determinations would be needed so as to understand long term body burden characteristics. If a population has stable MeHg body burden levels, or if long term averages can be identified, biomarkers such as toenail could be used to identify causal association even though the biomarker may be chronologically removed from disease manifestation. These determinants are relevant to the relationship between ongoing exposures and endpoints resulting from chronic exposure (i.e. cardiovascular disease) but would not be as applicable to circumstances associated with the population consisting of women of child bearing age. Long term variability will not be as relevant for gestational exposure as present day understanding suggests a finite window of susceptibility for women during pregnancy may exist that can result in deleterious effects; possibly even from a single exposure resulting in a large bolus dose at a critical time period during fetal development.

A limitation of this work is that the intra-individual temporal variability observed in toenail-Hg levels, even though only two fold, could be a factor leading to imprecision in exposure measurement. Estimates of imprecision for biomarker parameters such as hair and blood that can impact dose estimations in dose–response models have already been provided and the toenail compartment as a reservoir reflecting MeHg body burden may include similar imprecision [39]. When investigating dose–response relationships, unbiased imprecision can lead to an underestimation of the relationship between dose and response. In the present work, where two toxicokinetic compartments are being compared, the imprecision will not lead to bias but will provide for a random error effect.

Chronologically matching hair with toenails provided for high correlation and yielded a slope of 2.79 based on a regression model (Table 1). Data that were obtained from samples collected at the same point in time (i.e. during a clinic visit) and thus not chronologically matched with respect to exposure period were also shown to correlate and were similar to results found in other studies comparing data from biomarkers collected at the same time but not representing the same period of exposure (Table 1). Nonetheless, the chronologically matched ratio should be used to estimate Hg levels in the hair compartment based on toenail levels. The chronologically matched ratio would allow for estimating the corresponding Hg levels in hair that have been widely used as a biomarker for investigating multiple toxicological effects, including cardiovascular outcomes and, would allow for comparison across studies as the hair-Hg data set within the literature is robust.

For any given population, if the hair- and toenail-Hg ratios from chronologically matched and unmatched samples are in reasonable agreement, we may conclude that their body burdens are relatively stable over time. From the existing population data sets (Table 1), this reasoning would suggest that the non-occupationally exposed Japanese women [15], one group of non-occupationally exposed Finns [13] and possibly the control group from within the dentists study [26] may have stable body burden levels. However, even if the body burden in a population is found to be, on average, stable over a given time period, it cannot necessarily be assumed that such stability can be extrapolated to much longer time periods. Thus, attempts at identifying stability in body burden within a study population should be made if biomarkers chronologically removed from disease manifestation are to be used to investigate a dose response relationship.

## Conclusions

The data presented in this study will allow for an improved estimation of MeHg exposure based on the toenail compartment which is used as an exposure metric in studies examining the relationship between cardiovascular effects and MeHg exposure. A chronologically matched hair-Hg to toenail-Hg ratio has been identified within a population that has a MeHg body-burden level that is relatively stable over time. Intra-individual variation was less than two-fold, which may represent normal body burden fluctuation for individuals consuming elevated amounts of various fish species with different contaminant levels. The use of biomarker-based estimates reflecting body-burden levels that are chronologically distant from manifestation of deleterious outcome, or that are used as the sole indicator reflecting chronic exposure, need to be considered carefully when testing a hypothesis as individual body-burden levels may not remain constant across lengthy time periods such as a decade.

## Abbreviations

AMIBS: Arsenic Mercury Intake Biometric Study; EPA: Environmental Protection Agency; GM: Geometric Mean; Hg: Mercury; MeHg: MethylMercury; RfD: Reference Dose; US: United States; WA: Washington State.

## Competing interests

All authors declare they have no actual or potential competing financial or non-financial interests in this work.

## Authors’ contributions

TH conducted the hair-Hg and toenail-Hg analyses and provided the Supplemental Materials. AT was a significant contributor to almost all aspects of this project involving the Korean and Japanese populations. AMIBS would not have been as successful without her. AS aided in writing the manuscript. TMB contributed significantly to study design and data interpretation. EM aided in the writing of the manuscript and contributed to the acquisition of the data. All authors contributed substantially to the discussion of the data and their analyses, and provided editorial comments to the draft manuscript.

## Disclaimer

The contents of this manuscript are solely the responsibility of the authors and do not necessarily represent the official views or policies of the U.S. EPA, NIH/National Institute of Environmental Health, National Science Foundation, New Jersey Department of Environmental Protection, or Washington State Department of Health.

## Supplementary Material

Additional file 1 **Supplemental Material.** Provides additional text to description found in Methods section on hair-Hg representativeness for each hair sample. Click here for file
