# Supplementary material for: Chronologically matched toenail-Hg to hair-Hg ratio: temporal analysis within the Japanese community (U.S.)
Source: Environ Health. 2012 Oct 31;11:81. doi: 10.1186/1476-069X-11-81 (PMC3511224; doi:10.1186/1476-069X-11-81)
Supplement: Additional file 1 — Supplemental Material. Provides additional text to description found in Methods section on hair-Hg representativeness for each hair sample. [file 1476-069X-11-81-S1.doc]

**Supplemental Material**

An evaluation was conducted to assess the representativeness of hair-Hg data (for a specimen) in relation to the number of fibers analyzed. Relative standard deviations (RSDs) between 3.5 and 5.1% were obtained when 4 fibers were randomly selected (15 times) from a set of 20 where the Hg average (n=20) was 1.53 ng/mg (where three-significant figures are justified because each equal-length fiber was long enough to weigh to three-significant figures on a balance readable to 0.01 mg). Because there are 4,845 ways to select 4 items out of 20, all combinations were not computed, but the 4 fibers with the lowest Hg have an average Hg at 1.47 ng/mg, or 96% of the 1.53-ng/mg average above; and there is only a 0.02% chance (1/4845) of picking those 4 fibers. An RSD of 3.3% was obtained for a specimen with an average Hg of 2.39 ng/mg (for 5 sets of 4 fibers) while 20 other fibers showed a similar Hg average at 2.29 ng/mg. Using 6 fibers in consecutive-segment analyses for another donor, maximum-segment-Hg values at 4.18 and 4.38 ng/mg were obtained for specimens collected at two times, which illustrates that representativeness (using a few fibers) is reproducible when long hair is sampled at different times because the specimen with the new growth only showed the segment with the maximum Hg shifted along the fibers. Using hair specimens collected at three times from another donor, chronologically matched segments showed a 3.8% RSD for 10 fibers (average Hg 1.74 ng/mg).

Concerning our exposure-variation hypothesis [1], the biggest difference observed between adjacent 1.0-cm segments was 1.09 ng/mg (2.25 versus 1.16 ng/mg) while other fibers from this same specimen showed a 1.26- ng/mg change between the proximal 1.75-cm segment (1.89 ng/mg) and the adjacent 1.75-cm segment (0.63 ng/mg), where the latter comparison is a 3-fold change that crosses the 1.2- ng/mg hair criterion [considered the hair-Hg exposure equivalent of the methylmercury consumption Reference Dose (RfD)]. The differences in Hg between segments of the latter specimen cannot be attributed to any loss of Hg caused by permanent-waving, or straightening, treatments of the hair [such as the 30% loss described by Yamamoto and Suzuki [2], Ohno et al. [3]] because the 6-fiber segment with the maximum Hg for the newer growth specimen at 4.38 ng/mg is similar to the maximum-segment Hg at 4.18 ng/mg for the specimen obtained at the previous collection time from this donor, i.e., no evidence of any Hg loss from the specimens collected at the two times. The largest Hg difference found between adjacent-hair segments (of any length) was a 1.72-ng/mg increase for the 2.03-cm proximal segment versus the adjacent 2.03-cm segment (6.47 vs 4.75 ng/mg), which provides further evidence in support of our exposure-variation hypothesis.

Even when Hg differences in short adjacent segments of hair are less dramatic than >1 ng/mg, Hg differences in non-adjacent hair segments can demonstrate a lack of steady-state exposure, which is consistent with our exposure-variation hypothesis [1]. For example, four consecutive 2-cm segments from a specimen showed adjacent-segment Hg differences of 0.46, 0.39, and 0.45 ng/mg such that the accumulative effect is that the first segment has 1.30-ng/mg more Hg than the 4th segment (4.75 versus 3.45 ng/mg). Although 6-cm proximal hair segments from hair specimens obtained at three times from one donor suggested rather uniform Hg exposure (with respective values of 1.76, 1.82, and 1.97 ng/mg), sub-segments from the earliest specimen revealed values of 2.35 ng/mg, 1.82 ng/mg, and 1.31 ng/mg where the weighted average at 1.79 ng/mg is near the 1.76 ng/mg for the original proximal-segment measurement. Bartell et al. [4] have stated that "...mercury exposures are not likely to occur under steady-state conditions" and, more generally, that "...toxicant exposures rarely, if ever, occur under steady-state conditions." Some evidence for uniform Hg exposure for short periods has been observed in our data. While 14 consecutive 1-cm segments from one specimen varied in Hg from 0.868 to 1.74 ng/mg, the 7th through 12th segments only varied in Hg between 1.62 and 1.74 ng/mg (average 1.68 ng/mg with 2.9% RSD). Similarly, for consecutive 2.35-cm segments from another specimen where the Hg varied from 0.918 to 2.54 ng/mg across 8 segments, the four segments starting at 6.50 cm proximal vary from 1.49 to 1.91 ng/mg (average 1.71 ng/mg with 10% RSD). To conclude, both Hg representativeness and responsiveness (to dietary changes) can be achieved using segments from a few hair fibers (when weighing is done to 3-digit accuracy).

References

1. Tsuchiya A, Hinners TA, Krogstad F, White JW, Burbacher TM, Faustman EM, Mariën K: **Longitudinal mercury monitoring within the Japanese and Korean Communities (United States): Implications for exposure determination and public health protection.** *Environ Health Per* 2009, **117**:1760-1766.
2. Ohno T, Sakamoto M, Kurosawa T, Dakeishi M, Iwata T, Murata K: **Total mercury levels in hair, toenails, and urine among women free from occupational exposure and their relations to renal tubular function**. *Environ Res* 2007, **103**:191-197.
3. Yamamoto R, Suzuki T: **Effects of artificial hair-waving on hair mercury values**. *Int Arch Occup Environ Health* 1978, **42**:1-9.
4. Bartell SM, Griffith WC, Faustman EM: **Temporal error in biomarker-based mean exposure estimates for individuals**. *J Expo Sci Environ Epidemiol* 2004, **14**:173-179.
